# Comparison of Serological Biomarkers in Rheumatoid Arthritis and Their Combination to Improve Diagnostic Performance

**DOI:** 10.3389/fimmu.2018.01113

**Published:** 2018-06-06

**Authors:** Laura Martinez-Prat, Michael J. Nissen, Céline Lamacchia, Chelsea Bentow, Laura Cesana, Pascale Roux-Lombard, Cem Gabay, Michael Mahler

**Affiliations:** ^1^Research and Development, Inova Diagnostics, Inc., San Diego, CA, United States; ^2^Division of Rheumatology, Geneva University Hospitals (HUG), Geneva, Switzerland; ^3^Experimental Laboratory of Immunological and Rheumatologic Researches, Istituto Auxologico Italiano, Milan, Italy; ^4^Division of Immunology and Allergy, Department of Internal Medicine Specialties, Geneva University Hospital (HUG), Geneva, Switzerland

**Keywords:** rheumatoid arthritis, RA, diagnosis, anti-citrullinated peptide/protein antibodies, CCP3, CCP2, rheumatoid factor, RF

## Abstract

**Introduction:**

The diagnosis of rheumatoid arthritis (RA) is based on a combined approach that includes serological markers such as rheumatoid factor (RF) and anti-citrullinated peptide/protein antibodies (ACPA). The goal of this study was to evaluate the clinical performance of several RF and ACPA immunoassays for the diagnosis of RA, as well as the diagnostic value of a combinatory approach with these markers.

**Methods:**

The study cohort included 1,655 patients from the Swiss Clinical Quality Management registry with sera from 968 patients with RA and 687 disease controls, including patients with axial spondyloarthritis (*n* = 450) and psoriatic arthritis (*n* = 237). ACPA were determined by anti-CCP2 IgG enzyme-linked immunosorbent assay (ELISA), QUANTA Flash^®^ CCP3 IgG [chemiluminescent immunoassay (CIA)], and QUANTA Lite^®^ CCP3 IgG ELISA. RF was determined by ELISA (QUANTA Lite^®^ RF IgM, RF IgA, and RF IgG) and with two research use only CIAs (QUANTA Flash^®^ RF IgM and RF IgA).

**Results:**

All three ACPA assays showed good discrimination between RA patients and controls and good clinical performance. Overall, CCP3 performed better than CCP2. More pronounced differences were observed between the RF assays. We observed that CIA platforms for both RF IgM and RF IgA showed better performance than the ELISA platforms. Excellent and good total agreements were found between ELISA and CIA for CCP3 (total agreement 95.3%, kappa = 0.90), and between CCP2 and CCP3 ELISA (total agreement 86.6%, kappa = 0.73), respectively. RF IgM CIA and ELISA had a good qualitative agreement (86.5%, kappa = 0.73); RF IgA CIA and ELISA showed a moderate total agreement (78.5%, kappa = 0.53). When combinatory analyses were performed, the likelihood of RA increased with dual positivity and triple positivity and combining different markers resulted in higher odds ratio than the individual markers in all cases.

**Conclusion:**

ACPA and RF showed good clinical performance in this large Swiss cohort of RA patients and controls. Overall, the performance of CCP3 was superior to CCP2. The combination of these biomarkers in an interval model represents a potential tool for the diagnosis of RA patients.

## Introduction

Rheumatoid arthritis (RA) is a chronic autoimmune disease that is characterized by pain, inflammation, and joint destruction and affects up to 1.0% of the general population (1, 2). Early diagnosis and treatment in RA is crucial as it can prevent disease progression and irreversible joint damage (3–5). RA diagnosis is based on a combined approach that consists of history acquisition, clinical examination, imaging modalities, and testing of acute-phase and serological markers such as rheumatoid factor (RF) and anti-citrullinated peptide/protein antibodies (ACPA) (2).

Rheumatoid factor IgM, the main isotype identified by RF assays, is found in approximately 70–80% of patients with confirmed RA (6–8). In addition, elevated levels of RF IgA and IgG have been reported in patients with RA (9, 10). Studies suggest that elevated levels of RF IgG are highly specific for RA diagnosis (10, 11). It has been proposed that the detection of all three RF isotypes improves the specificity and predictive value of RF testing (12).

A caveat of RF testing is its low specificity and that it can be found in patients with infections and other autoimmune diseases, as well as in a proportion of healthy individuals (13), with rates between 10 and 25% in elderly patients without RA (14). Despite this, RF was the only serological marker included in the 1987 American College of Rheumatology (ACR) classification criteria.

In 1998, the presence of autoantibodies specific to citrulline-containing antigens was reported in RA patients (15). Clinical studies confirmed that ACPA were more specific than RF for a diagnosis of RA. ACPA have a higher sensitivity than RF in early RA, an improved specificity, and good positive predictive value (16). Consequently, ACPA were included in the 2010 revised ACR/European League Against Rheumatism (EULAR) criteria for RA. These new classification criteria differentiate between low- and high-positive ACPA and RF levels, with greater weight given to higher autoantibody levels (2).

Anti-citrullinated peptide/protein antibodies are generally detected using anti-cyclic citrullinated peptide (CCP) antibody assays (17). The first generation of the CCP test used a peptide derived from the filaggrin protein as the antigen. The second and third generation CCP (CCP2 and CCP3, respectively) are no longer based on the filaggrin-derived native sequences, but on peptides specifically designed and optimized (mimotypes) to detect ACPA. These improvements enhance the immunoreactivity of the citrulline-containing epitope (18–20). Serum samples from a subset of RA patients that report negative for the anti-CCP2 assay (second generation) can react to other citrullinated proteins (21–24). This suggests that there are additional epitopes that are not present in the second generation CCP antigen sequence. The third generation CCP antigen was developed by testing a large number of RA patients and control subjects on various citrullinated peptides (25, 26).

Anti-citrullinated peptide/protein antibodies and RF are widely used aids in the diagnosis and classification of RA. The combination of results from these markers might provide further value in the management of RA. Furthermore, it was recently demonstrated that the combined presence of RF IgM and ACPA mediates increased production of pro-inflammatory cytokines *in vitro* and is associated with elevated systemic inflammation and disease activity in RA (27, 28).

Nevertheless, many patients are seronegative for ACPA and RF, and there is a need for novel serological biomarkers to help close this serological gap (29) and improvement of early diagnosis, classification of RA subtypes, and patient stratification.

Enzyme-linked immunosorbent assay (ELISA) and bead-based chemiluminescent immunoassays (CIAs) are two frequently used tests for the quantification of ACPA and RF. Several differences between these two platforms have been described (30). One of those differences includes potentially improved sensitivity due to the larger surface binding area of the bead-based assay. Although it is possible that laboratories use both technologies, ELISA and CIA, the majority of laboratories prefer to run all tests on the same platform when possible, due to increased efficiency. Therefore, we decided to focus on the combinations of assays on the same platform.

This study aimed to evaluate the clinical performance of several immunoassays (plate-based ELISA- and bead-based CIA-) for the detection of RF and ACPA as aids in diagnosis of RA, as well as the diagnostic value of an approach based on combinations of outcomes of these serological biomarkers.

## Materials and Methods

### Patients and Sera

All patients included in this study originated from the national Swiss registry established in 1997, the Swiss Clinical Quality Management (SCQM), which collects data from patients with inflammatory rheumatic diseases (http://scqm.ch) (31, 32). The registry longitudinally collects clinical, safety, and radiological data from patients with RA, axial spondyloarthritis (axSpA), and psoriatic arthritis (PsA). The diagnosis is based on the opinion of board-certified rheumatologists. In 2010, a biobank situated at the Department of Genetic and Laboratory Medicine of the University Hospital in Geneva (HUG) was established. This biobank includes serum samples of patients participating in the SCQM registry. Participation with the SCQM registry is on a purely voluntary basis. All patients provided signed informed consent prior to inclusion in the SCQM registry. An additional separate signed informed consent following Institutional and State regulations was collected for the biobank prior to blood acquisition. All biological samples are stored and used anonymously in this study. The study protocol received approval of the local ethics commission of the University Hospital of Geneva (protocol 10-089) and of the SCQM Biobank Scientific Advisory Board. All serological samples available at the time of the study were included in the analysis. The cohort comprised sera collected from 1,655 patients (968 RA and 687 controls). The controls included patients with axSpA (*n* = 450) and PsA (*n* = 237). A summary of the patients’ characteristics can be found in Table 1. For the majority of patients in the registry, the classification criteria for the three different diseases were available. For axSpA, the Assessment of Spondyloarthritis International Society criteria were utilized (33) and for PsA the classification criteria for psoriatic arthritis were utilized (34). The majority of patients classified as negative for the correspondent disease criteria were as a result of missing data required to confirm the criteria.

**Table 1:** Summary of patients’ characteristics at baseline.

	Total	RA		axSpA		PsA	
	All	All	ACR-EULAR+	All	ASAS+	All	CASPAR+
				
	*N* = 1,655	*N* = 968	*N* = 780	*N* = 450	*N* = 316	*N* = 237	*N* = 185
Age (SD)	49.2 (13.7)	53.1 (13.2)	52.7 (12.8)	41.1 (12.2)	38.6 (11.1)	48.3 (12.1)	48.0 (12.2)
Gender (% male)	39.4	25.6	22.9	59.3	63.1	57.8	58.4
Disease duration (median years) (IQR)	5.65 (2.1, 13.2)	4.56 (1.6, 10.6)	4.62 (1.6, 10.9)	9.3 (4.1, 18.1)	10.7 (4.7, 19.1)	5.3 (1.8, 13.1)	5.0 (2.0, 12.1)
BMI (median) (IQR)	25.3 (22.3, 29.0)	25.2 (22.2, 29.1)	25.1 (22.1, 29.0)	24.9 (22.4, 28.1)	24.8 (22.4, 27.9)	26.7 (23.7, 29.9)	26.9 (23.9, 30.1)
Smoker (% actual or past)	58.0	57.3	58.0	57.2	60.5	63.1	64.4
Alcohol consumption (%)	72.5	70.2	69.3	–	–	82.3	82.2
Higher education (%)	57.1	57.9	58.5	57.1	58.0	54.4	54.1
CRP	9.9	10.4	11.1	9.9	10.5	7.8	7.9
Raised CRP or ESR (%)	43.5	51.0	54.9	35.1	37.3	29.1	29.7
Physician global score	3.2 (2.2)	3.3 (2.3)	3.6 (2.3)	3.1 (2.1)	3.1 (2.0)	3.2 (2.3)	3.3 (2.3)
DAS28 (SD)	–	3.8 (1.6)	4.0 (1.5)	–	–	2.8 (1.4)	2.7 (1.5)
Erosive radiographic changes (%)	–	59.6	65.0	–	–	–	–
HAQ (median, IQR)	–	0.75 (0.25, 1.375)	0.875 (0.375, 1.375)	–	–	0.5 (0.125, 1)	0.5 (0.125, 1)
RADAI	–	3.63	3.82	–	–	–	–
BASDAI	–	–	–	4.43 (2.4)	4.4 (2.4)	–	–
BASFI	–	–	–	3.1 (2.6)	3.0 (2.6)	–	–
BASMI	–	–	–	2.0 (1.8)	1.9 (1.9)	–	–
ASDAS-CRP	–	–	–	2.68 (1.2)	2.75 (1.2)	–	–

*Mean values (±SD) unless otherwise indicated*.

*RA, rheumatoid arthritis; axSpA, axial spondyloarthritis; ASAS, Assessment of Spondyloarthritis International Society; PsA, psoriatic arthritis; CASPAR, classification criteria for psoriatic arthritis; IQR, inter quartile range; CRP, C-reactive protein; ESR, erythrocyte sedimentation rate; DAS28, disease activity score 28; HAQ, health assessment questionnaire; RADAI, rheumatoid arthritis disease activity index; BASDAI, Bath ankylosing spondylitis disease activity index; BASFI, Bath ankylosing spondylitis functional index; BASMI, Bath ankylosing spondylitis metrology index; ASDAS-CRP, ankylosing spondylitis disease activity score-C-reactive protein; ACR, American College of Rheumatology; EULAR, European League Against Rheumatism*.

### Immunoassays

Anti-citrullinated peptide/protein antibodies were determined by anti-CCP2 IgG ELISA (Euro Diagnostica, Malmö, Sweden), QUANTA Flash^®^ CCP3 IgG CIAs (Inova Diagnostics, San Diego, CA, USA), and QUANTA Lite^®^ CCP3 IgG ELISA (Inova). RF was determined by ELISA (QUANTA Lite RF IgM, RF IgA, and RF IgG, all Inova), and by QUANTA Flash RF IgM and RF IgA, two CIAs [research use only (RUO), both Inova] designed for the BIO-FLASH^®^ Instrument (Biokit s.a., Barcelona, Spain). The principles and protocols of the BIO-FLASH assay system have been previously described (30). The QUANTA Flash RF assays are novel CIA that use rabbit IgG as antigen, coated onto paramagnetic beads. Results obtained with CCP2 ELISA were expressed in U/mL. Results measured with CCP3 CIA IgG assay were expressed in chemiluminescent units. Results obtained with RF CIA IgM and IgA were expressed in relative light units. Results with the CCP3 IgG and RF IgM, IgA, and IgG ELISAs were expressed in Units. All tests were performed according to the manufacturers’ instructions for the commercially available assays and to research protocols for the RUO assays. Cutoff values recommend in the instruction for use were applied. Preliminary cutoff values for the RF IgM and IgA RUO CIA were established using the 95% CI of the reference limit at ≥95% in a selected cohort of samples (*n* = 191) including samples from patients suffering from systemic lupus erythematosus (*n* = 9), vasculitis (*n* = 5), antiphospholipid syndrome (*n* = 12), celiac disease (*n* = 8), infectious diseases (*n* = 27) including HBV (*n* = 5), HCV (*n* = 6), HIV (*n* = 9), and syphilis (*n* = 7), apparently healthy individuals (*n* = 117), and samples with anti-nuclear antibodies (*n* = 13). A summary of the characteristics of the different assays can be found in Table S1 in Supplementary Material.

### Statistical Evaluation

**Figure 1:**
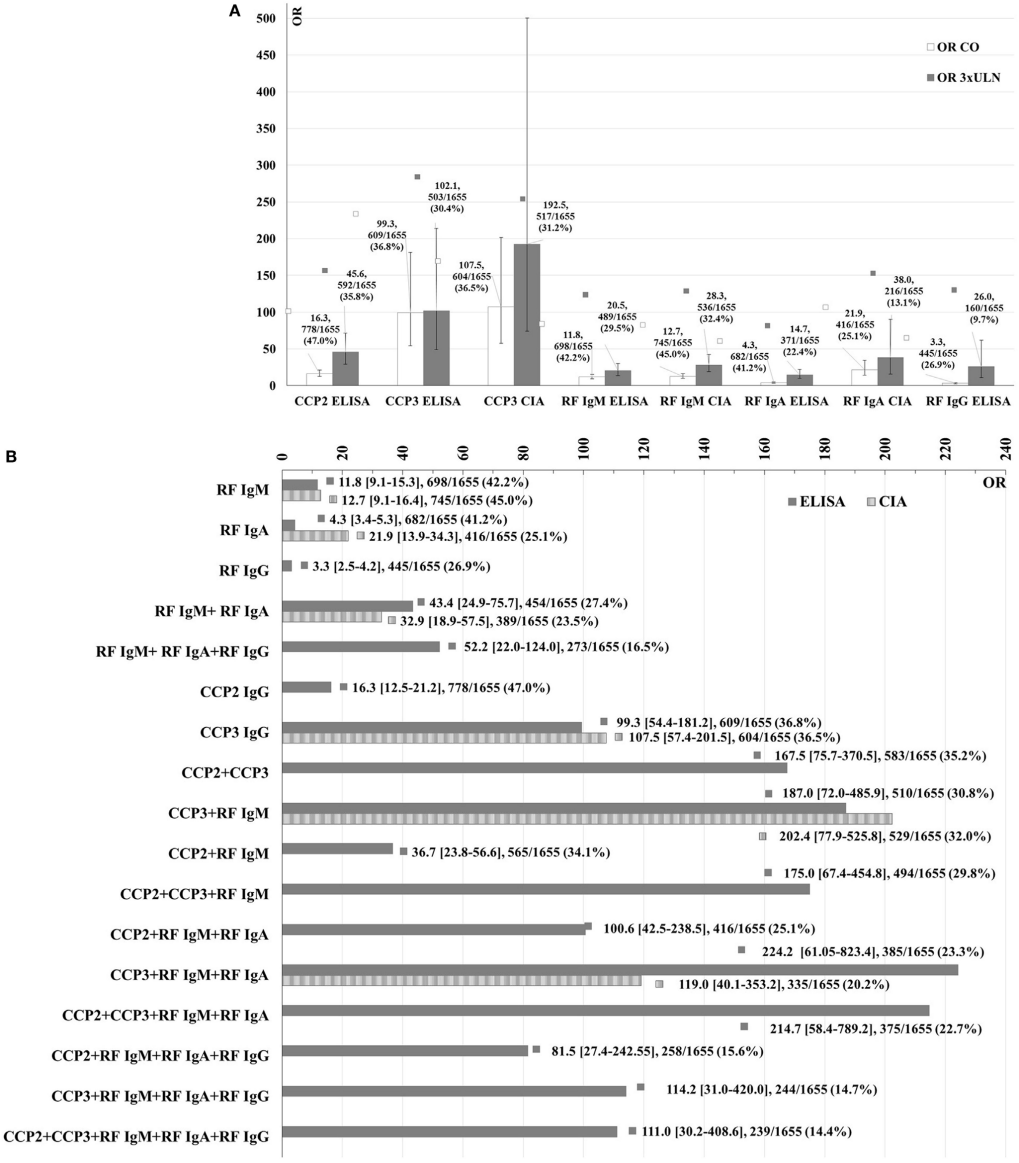
Association of single markers with RA [OR] on the ELISA and CIA platforms at the manufacturers cutoff or preliminary cutoffs for the research use only assays versus at the 3× ULN **(A)** and association of single markers and combinations of markers with RA (OR) on ELISA (solid) and CIA (dashed) at the cutoffs **(B)**. OR values, 95% CI, number of patients positive for each condition, and % over the total number of patients included in the study are shown.

All statistical analyses were performed by *Analyse-it*^®^ for Excel method evaluation software (version 4.81.1; Leeds, UK) and the Python Scipy module. Receiver-operating characteristic (ROC) analyses were carried out to analyze the discrimination between RA patients and controls. The ROC curves were plotted, with the area under the curve (AUC) as an indicator of the diagnostic value. Sensitivities, specificities, and odds ratios (OR) were calculated based on the manufacturer’s cutoff for the commercially available assays and on the preliminary cutoffs for the CIA RUO assays, as well as at the 3× upper limit of normal (ULN) for each assay (Figure 1A). Sensitivities and OR were also calculated based on the 95% specificity cutoffs. A *t*-test and Benjamini–Hochberg correction, false discovery rate set at 5%, were run for the individual markers. Likelihood plots were generated to compare the change of OR at the assay cutoff point versus the 3× ULN for the seven assays and also, to note if any optimal cutoffs with higher OR could be observed. Method comparison between platforms for the markers available on ELISA and CIA was performed. Combinations of markers were created at the manufacturer’s cut-offs and at 3× ULN (Figure 1B). ORs and the number of patients within each condition were calculated. Differences between likelihood ratios for the combinations of markers were calculated using BDTcomparator as described previously (35, 36). For these differences, *p*-values <0.05 were considered significant.

**Figure 2:**
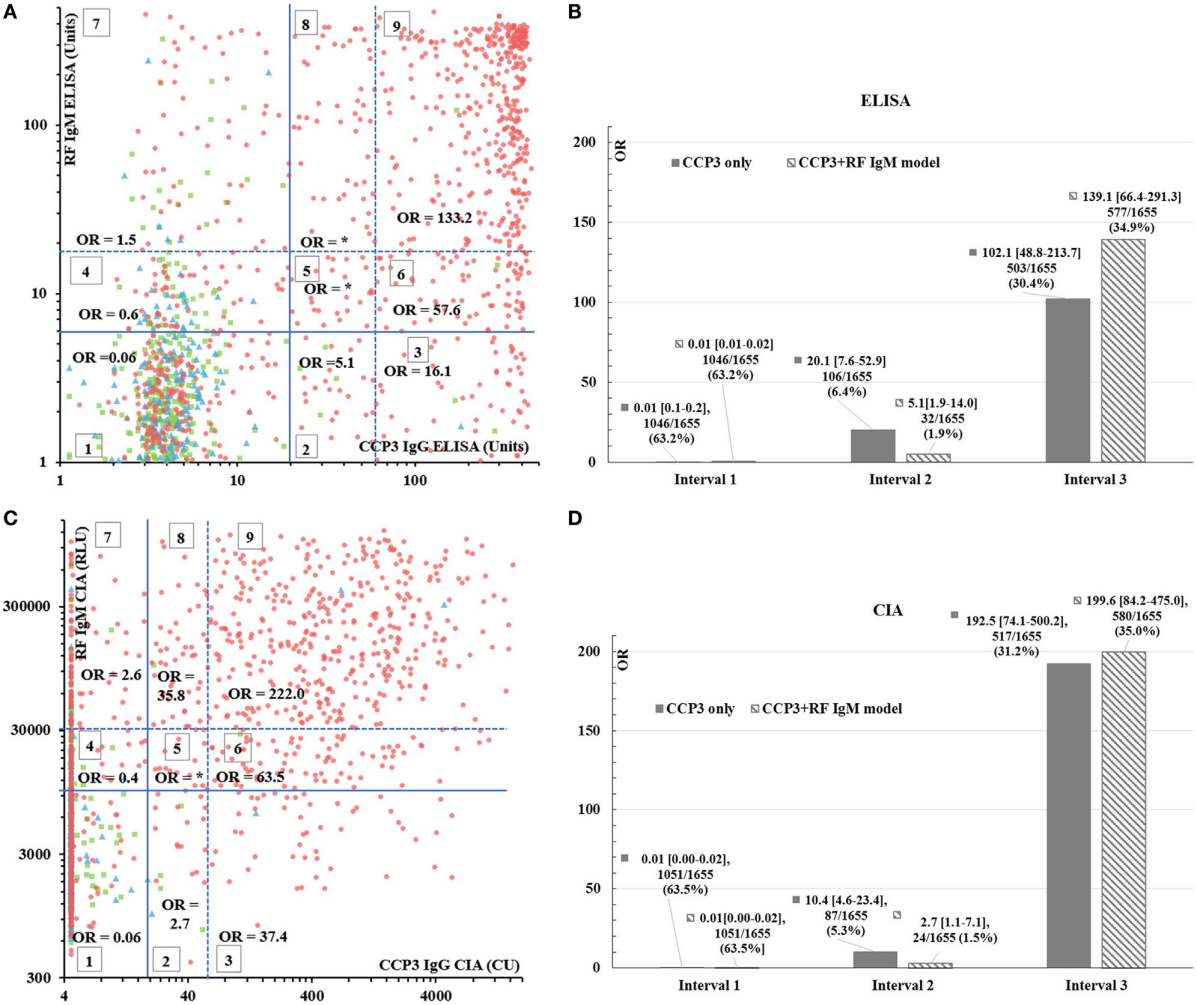
RF IgM and CCP3 combinatory model. Panels **(A,C)** represent the scatter plots for the RF IgM and CCP3 combination on ELISA **(A)** and CIA **(C)**. The red dots represent patients with RA, the green squares are patients with axSpA and the blue triangles show patients with PsA. The lines sectioning the graphs into nine groups represent the manufacturer’s cutoffs (solid line) or the preliminary cutoffs for the RUO assay and the 3× ULN (dashed). The different sections of the graph are indicated by numbers (1–9). The *x* and *y*-axes are shown in logarithmic scale. Panels **(B,D)** show the OR, 95% CI, number and percentage of patients for the individual CCP3 assays (solid) versus the combined model of CCP3 and RF IgM (dashed) in the three intervals on ELISA **(B)** and CIA. *Note: OR could not be calculated since no controls were found in those groups. Abbreviations: CU, chemiluminescent units; RLU, relative light units; CO, cutoff; ULN, upper limit of normal; OR, odds ratio; RF, rheumatoid factor; CIA, chemiluminescent immunoassay; ELISA, enzyme-linked immunosorbent assay; RA, rheumatoid arthritis; axSpA, axial spondyloarthritis; RUO, research use only.

To further investigate the model based on the RF IgM and CCP3 combination, scatter plots were created for the results of these two markers on each platform (Figure 2A for ELISA and Figure 2C for CIA). The plots were sectioned into nine groups using the manufacturer’s cutoffs or the preliminary cutoffs for the RUO assay and the 3× ULN. The OR was calculated in each section. In addition, based on clinical significance, several combinations of sections were created for both platforms, assuming three intervals of patients: patients with a low likelihood of RA (interval I), patients within an area of uncertainty (interval II), and patients with a high likelihood of RA (interval III). The OR and the number of patients within each combination were calculated. The selection of the best combination was made based on two factors: the OR for RA and the number of patients correctly classified. These combinations were compared with the individual performance of CCP3 below the cutoff (interval I), between the cutoff and the 3× ULN (interval II), and above the 3× ULN (interval III).

## Results

### Comparison of the Clinical Performance

All three ACPA assays (CCP2 ELISA and CCP3 ELISA and CIA) showed good discrimination between RA patients and controls, with AUC of 0.82, 0.83, and 0.82, respectively. At the cutoff values provided by the manufacturer, the CCP2 ELISA showed a high sensitivity (71.1%) and a moderately high specificity (86.9%) with a corresponding OR of 16.3 (95% CI 12.5–21.1). The two CCP3 assays showed lower sensitivities (61.8% for ELISA and 61.4% for CIA), but significantly higher specificities (98.4 and 98.5%, respectively), resulting in much higher predictive values, with OR of 99.3 (95% CI 54.4–181.2) and 107.5 (95% CI 57.4–201.5), respectively. The clinical performance characteristics for all assays are summarized in Table 2.

**Table 2:** Summary of clinical performance characteristics of the assays used in this study.

	CCP2 IgG ELISA	CCP3 IgG ELISA	CCP3 IgG CIA	RF IgM ELISA	RF IgM CIA	RF IgA ELISA	RF IgA CIA	RF IgG ELISA
Cutoff	25 U/mL	20 U	20 CU	6 U	10,344 RLU	6 U	7,425 RLU	6 U
# Positive samples/total # samples (%)	778/1,655 (47.0)	609/1,655 (36.8)	604/1,655 (36.5)	698/1,655 (42.2)	745/1,655 (45.0)	682/1,655 (41.2)	416/1,655 (25.1)	445/1,655 (26.9)
# Positive RA samples/total # RA samples (%)	688/968 (71.1)	598/968 (61.8)	594/968 (61.4)	611/698 (63.1)	650/968 (67.1)	530/968 (54.8)	395/968 (40.8)	345/968 (35.6)
# Positive samples/total # axSpA samples (%)	59/450 (13.1)	8/450 (1.8)	6/450 (1.3)	56/450 (12.4)	60/450 (13.3)	103/450 (22.9)	14/450 (3.1)	68/450 (15.1)
# Positive PA samples/total # PsA samples (%)	31/237 (13.1)	3/237 (1.3)	4/237 (1.7)	31/237 (13.1)	35/237 (14.8)	49/237 (20.7)	7/237 (3.0)	32/237 (13.5)
Sensitivity (%)	71.1	61.8	61.4	63.1	67.1	54.8	40.8	35.6
Specificity (%)	86.9	98.4	98.5	87.3	86.2	77.9	96.9	85.4
OR (95% CI)	16.3 (12.5–21.2)	99.3 (54.4–181.2)	107.5 (57.4–201.5)	11.8 (9.1–15.3)	12.7 (9.9–16.4)	4.3 (3.4–5.3)	21.9 (13.9–34.3)	3.3 (2.5–4.2)
AUC (95% CI)	0.82 (0.80–0.84)	0.83 (0.81–0.85)	0.82 (0.81–0.84)	0.79 (0.77–0.81)	0.82 (0.80–0.84)	0.70 (0.67–0.72)	0.79 (0.77–0.81)	0.61 (0.59–0.64)
Cutoff for 95% specificity	53.0	8.3	6.6	14.6	24,767	14.2	5,252	8.3
Sensitivity at 95% specificity	61.3	68.1	66.1	50.2	55.0	39.7	48.1	27.6
OR at 95% specificity (95% CI)	29.7 (20.7–42.7)	39.9 (27.7–57.5)	36.5 (25.4–52.6)	18.8 (13.1–27.0)	22.1 (15.4–31.5)	12.6 (8.7–18.2)	17.3 (12.1–24.8)	7.2 (5.0–10.4)
Cutoff 3× ULN	75	60	60	18	31,032	18	22,275	18
Sensitivity at 3× ULN (%)	59.0	51.2	53.0	47.5	52.7	35.7	21.8	16.0
OR at 3× ULN (95% CI)	45.6 (29.1–71.5)	102.1 (48.8–213.7)	192.5 (74.1–500.2)	20.5 (13.9–30.4)	28.3 (18.8–42.6)	14.7 (9.7–22.4)	38.0 (16.0–90.4)	26.0 (10.9–62.1)
*p*-Value	7.31e−29	1.36e−105	2.87e−25	8.09e−58	1.23e−48	4.44e−36	2.53e−13	2.29e−15
FDR	True	True	True	True	True	True	True	True

*CCP, cyclic citrullinated peptide; RF, rheumatoid factor; CIA, chemiluminescent immunoassay; RUO, research use only; CU, chemiluminescent units; RLU, relative light units; OR, odds ratio; AUC, area under the curve; ULN, upper limit of normal; FDR, false discovery rate; RA, rheumatoid arthritis; axSpA, axial spondyloarthritis; ELISA, enzyme-linked immunosorbent assay*.

For RF, more pronounced differences between assays were observed. The AUC derived from ROC analysis ranged from 0.61 (RF IgG ELISA) to 0.82 (RF IgM CIA). At the cutoffs provided by the manufacturer for the ELISAs and at the preliminary cutoffs established for the CIAs, the sensitivities ranged from 35.6% (RF IgG ELISA) to 67.1% (RF IgM CIA), and the specificities were between 77.9% (RF IgA ELISA) and 96.9% (RF IgA CIA). The RF IgM CIA was the most sensitive, while the RF IgA CIA was the assay with the highest specificity. The RF IgG ELISA showed the lowest discrimination value (AUC = 0.61), with the lowest sensitivity at the recommended cutoff (35.6%). Differences were also observed between the predictive values of the RF assays, with OR that ranged from 3.3 (95% CI 2.5–4.2) for the RF IgG ELISA to 21.9 (95% CI 13.9–34.3) for RF IgA CIA. Interestingly, the OR for the RF IgA CIA was more than five times higher than the OR for the ELISA of this RF isotype (OR = 4.3, 95% CI 3.4–5.3). Similar results were obtained for both ACPA and RF when analyses were performed only including the patients for which diagnostic criteria was available (data not shown).

When all assays were compared at the same specificity (95%), the sensitivities varied from 27.6% (RF IgG ELISA) to 68.1% (CCP3 IgG CIA). As results greater than 3× ULN carry more clinical weight for the diagnosis and classification of RA as seen in the 2010 ACR/EULAR classification criteria (2), the clinical performance at the 3× ULN was also analyzed. At this threshold, the sensitivities ranged from 16.0% (RF IgG ELISA) to 59.0% (CCP2 ELISA) and the OR varied from 14.7 (95% CI 9.7–22.4, RF IgA ELISA) to 192.5 (95% CI 74.1–500.2, CCP3 IgG CIA).

In the RF IgM and CCP3 IgG negative population based on ELISA (*n* = 858), the RF IgA ELISA had an 18.5% sensitivity with 76.9% specificity and an OR of 0.8 (95% CI 0.5–1.1) and the RF IgG ELISA reported a 10.6% sensitivity, an 88.0% specificity and OR of 0.9 (95% 0.5–1.4). In the RF IgM and CCP3 IgG seronegative population defined by CIA (*n* = 835), the RF IgA showed a 5.2% sensitivity with a 98.6% specificity and an OR of 4.0 (95% CI 1.7–9.5).

To investigate if the combination of both ACPA tests with different citrullinated antigens (CCP2 and CCP3) provides better clinical performance, we looked at the performance of the CCP3 assay in the CCP2 negative population and vice versa. In the CCP2 negative population (*n* = 877), the CCP3 ELISA reported a sensitivity of 7.5%, a 99.2% specificity, and an OR of 9.6 (95% CI 3.7–24.9) and the CCP3 CIA had a 6.4% sensitivity with a 99.5% specificity and OR of 13.6 (95% CI 4.2–43.6). On the other hand, the CCP2 ELISA had a 30.0% sensitivity with an 87.6% specificity in the CCP3 ELISA negative population (*n* = 1,046) and a 29.9% sensitivity and 87.7% specificity in the CCP3 CIA negative population (*n* = 1,051), with OR of 3.0 (95% CI 2.2–4.2) and 3.1 (95% CI 2.2–4.2), respectively.

Likelihood plots were generated to compare the OR at the assay cutoff point versus the 3× ULN for the different assays and also, to note if any optimal cutoffs with higher OR could be observed (see Figure S1 in Supplementary Material). The association of single markers with RA on the CIA and ELISA platforms at the cutoffs versus at the 3× ULN was also analyzed (Figure 1A). As expected, higher OR were observed at the 3× ULN than at the cutoffs for all markers.

### Agreement Between Different Methods

An agreement greater than 95% was considered excellent, between 80 and 95% was good and between 50 and 80% was moderate. Excellent qualitative agreement was found between ELISA and CIA for CCP3, with a total agreement of 95.3% (95% CI 94.2–96.3%) and a kappa of 0.90 (95% CI 0.88–0.92). When the CCP2 and CCP3 ELISAs were compared, a good total agreement (86.6%, 95% CI 84.9–88.2%), moderate positive agreement (74.9%, 95% CI 71.8–77.9%), and excellent negative agreement (97.0%, 95% CI 95.7–98.0%) were observed and a kappa of 0.73 (95% CI 0.70–0.76) was found. The RF IgM CIA and ELISA showed a good qualitative agreement, with a total agreement of 86.5% (95% CI 84.8–88.1%), a positive agreement of 87.4% (95% CI 84.7–89.7%), a negative agreement of 85.9% (95% CI 83.5–88.0%), and a kappa of 0.73 (95% CI 0.69–0.76). For the RF IgA assays, although the negative agreement was high (95.4%, 95% CI 93.9–96.5%), the positive agreement was moderate (54.4%, 95% CI 50.6–58.1%) resulting in a moderately high total agreement (78.5%, 95% CI 76.4–80.4%) (kappa = 0.53, 95% CI 0.49–0.57). A summary of these agreement analyses can be found in Table 3.

**Table 3:** Agreement between the CCP2 and CCP3 ELISA assays (A), and between ELISA and CIA for CCP3 (B), RF IgM (C) and RF IgA (D).

Kappa = 0.73 (95% CI 0.70–0.76)		CCP3 IgG ELISA			Percent agreement (95% confidence interval)	
			
		Negative	Positive	Total		
**CCP2 IgG ELISA**	**Negative**	851	26	877	Neg. agreement	97.0% (95.7–98.0%)
	**Positive**	195	583	778	Pos. agreement	74.9% (71.8–77.9%)
	**Total**	1,046	609	1,655	Total agreement	86.6% (84.9–88.2%)

**Kappa = 0.90 (95% CI 0.88–0.92)**		**CCP3 IgG CIA**			**Percent agreement (95% confidence interval)**	
			
		**Negative**	**Positive**	**Total**		

**CCP3 IgG ELISA**	**Negative**	1,010	36	1,046	Neg. agreement	96.6% (95.3–97.5%)
	**Positive**	41	568	609	Pos. agreement	93.3% (91.0–95.0%)
	**Total**	1,051	604	1,655	Total agreement	95.3% (94.2–96.3%)
**Kappa = 0.73 (95% CI 0.69–0.76)**		**RF IgM CIA**			**Percent agreement (95% confidence interval)**	
	
		**Negative**	**Positive**	**Total**

**RF IgM ELISA**	**Negative**	822	135	957	Neg. agreement	85.9% (83.5–88.0%)
	**Positive**	88	610	698	Pos. agreement	87.4% (84.7–89.7%)
	**Total**	910	745	1,655	Total agreement	86.5% (84.8–88.1%)

**Kappa = 0.53 (95% CI 0.49–0.57)**		**RF IgA CIA**			**Percent agreement (95% confidence interval)**	
	
		**Negative**	**Positive**	**Total**		

**RF IgA ELISA**	**Negative**	928	45	973	Neg. agreement	95.4% (93.9–96.5%)
	**Positive**	311	371	682	Pos. agreement	54.4% (50.6–58.1%)
	**Total**	1,239	416	1,655	Total agreement	78.5% (76.4–80.4%)

*CCP, cyclic citrullinated peptide; RF, rheumatoid factor; CIA, chemiluminescent immunoassay; ELISA, enzyme-linked immunosorbent assay*.

### Combinations of Markers

When combinatory analyses were performed, the likelihood of RA increased with dual and triple positivity. Combining different markers resulted in higher OR than the individual markers in all cases (Figure 1B). Nevertheless, 95% CI of the OR overlapped in some cases indicating that the differences may not be clinically significant.

The predictive value of the RF test was increased by the detection of multiple isotypes. Combining ACPA and RF IgM resulted in higher OR than the individual markers. Differences were observed between the combinations that included CCP2 and/or CCP3 ELISA assays. The combination of CCP3 and RF IgM resulted in a higher OR (OR = 187.0, 95% CI 72.0–485.9) than in the combination of CCP2 with RF IgM (OR = 36.7, 95% CI 23.8–56.6). The triple combination of CCP2, RF IgM, and CCP3 IgG on ELISA resulted in a lower OR (OR = 175.0, 95% CI 67.4–454.8). Out of all the combinations analyzed, the highest OR was observed for CCP3 IgG, RF IgM, and RF IgA on ELISA (OR = 224.2, 95% CI 61.0–823.4), followed by CCP2, CCP3, RF IgM, and RF IgA on ELISA (OR = 214.7, 95% CI 58.4–789.2). The addition of RF IgG to these combinations resulted in a lower OR (see Figure 1B).

Receiver-operating characteristic analysis showed that the combination of CCP2 and CCP3 ELISA did not result in better discrimination between RA and controls than with individual CCP assays (AUC 0.79, 95% CI 0.78–0.81). Nevertheless, this combination had a higher specificity (99.1%) with a lower sensitivity (59.6%) and resulted in a higher OR (OR = 167.5, 95% CI 75.7–370.5).

In the model based on the RF IgM and CCP3 combination (Figure 2A for ELISA and Figure 2C for CIA), higher ORs were observed as the antibody levels of CCP3 and RF IgM increased. This is in accordance to the different weights assigned to different antibody levels in the 2010 ACR/EULAR classification criteria (2).

When the sections within the scatter plots for the RF IgM and CCP3 models were combined, for both platforms, the combination with highest OR and better classification based on number of patients in each group was identified as the following: patients with a low likelihood of having RA were defined as those that were negative for both RF IgM and for anti-CCP3 and those with positive RF IgM but who were negative for anti-CCP3 (groups 1, 4, and 7 = interval I); patients within an area of uncertainty were defined as those who were negative for RF IgM and demonstrated anti-CCP3 levels between the cutoff and the 3× ULN (group 2 = interval II); and finally patients with a very high likelihood of having RA were defined as the remaining patients (groups 3, 5, 6, 8, and 9 = interval III). These combinations were compared with the individual performance of CCP3 below the cutoff (interval I), between the cutoff and the 3× ULN (interval II), and above the 3× ULN (interval III) (Figures 2B,D). A higher number of patients could be correctly classified with the combinatory models than with the individual CCP3 markers.

## Discussion

Anti-citrullinated peptide/protein antibodies and RF are important serological markers for the diagnosis and classification of RA (2). RF was the first well-known marker in RA; however, it is known to have low specificity and to be present in various inflammatory diseases (37), which is consistent with the data in our study. When ELISA and CIA for the detection of RF IgM were compared, a high qualitative agreement was found. The RF IgM CIA showed a better discrimination between RA patients and the controls than the RF IgM ELISA, with a higher sensitivity and very similar specificity. Overall, the RF IgM CIA showed a better performance than the ELISA for the detection of these antibodies. This might be attributed to the technological advantages of the CIA as illustrated in a recent review article (30).

In addition to RF IgM, raised levels of RF IgG and IgA have been reported in patients with RA (10, 38, 39). At the preliminary cutoff, the RF IgA CIA showed a much higher specificity (the highest of all RF assays) compared with the ELISA. When the two assays were compared, a high negative agreement but a low positive agreement was found, resulting in a moderate total agreement. This could be due to the differences between the two platforms (30). While the RF IgA ELISA uses a polyclonal anti-human IgA antibody as conjugate, the CIA uses a monoclonal antibody for detection, which could explain the higher specificity of this platform compared with ELISA. It has been described that the detection of RF IgA in early disease suggests a poor prognosis and justifies more aggressive treatment (10, 38). However, most samples included in this study were derived from patients with established disease; therefore, the utility of RF IgA in early RA compared with advanced disease is outside the scope of this study. Nevertheless, our results suggest that the detection of RF IgA, when measured by CIA, can help to close the serological gap, due to some RA patients presenting RF IgA in the absence of RF IgM and ACPA.

Regarding RF IgG, the diagnostic value of this isotype in our cohort is not clear. RF IgG has a very low prevalence in RA patients, compared with the other two isotypes. In this study, the RF IgG ELISA showed a moderately high specificity similar to the RF IgM assays, with a very low sensitivity. Although this assay reported a very low sensitivity at the 3× ULN, the OR was moderately high. Since the performance of RF IgG on ELISA is limited, no RF IgG CIA was developed. However, RF IgG might have clinical utility in aspects other than the diagnosis, such as clinical association with vasculitis (11) or prediction for erosive disease and radiographic progression (40).

It has been suggested that the specificity and predictive value of the RF test is substantially increased by the detection of all three RF isotypes (12). Although we did not test for total RF, we saw that the dual and triple combinations of the RF isotypes showed higher OR than the individual markers, with the combination of the three isotypes measured by ELISA being the highest, among all the RF combinations analyzed.

Consistent with the results of several clinical studies, we observed that ACPA are more specific diagnostic markers than RF for RA, with the exception of RF IgA CIA in our study that also displayed a very high specificity (higher than CCP2). Significant differences between ACPA assays have been reported (41) and in our study, although the CCP3 CIA and ELISA showed equivalent performance and a high qualitative agreement, differences were observed between the CCP2 and the CCP3 assays, especially in the predictive value, with CCP3 outperforming CCP2.

Anti-citrullinated peptide/protein antibodies significantly improves the diagnosis of RA, especially in the RF negative population. Surprisingly, when analyzed at the 3× ULN, the CCP3 ELISA reported a very similar OR to that obtained at the manufacturers cutoff, as opposed to the CCP2 ELISA and the CCP3 CIA that showed a significant increase in OR at the 3× ULN. Out of all assays tested, the CCP3 CIA showed the highest OR at the assay cutoff as well as at the 3× ULN, confirming that the CCP3 CIA is a reliable test for the fully automated and rapid detection of ACPA, as previously described (20).

For the classification criteria, RF and ACPA carry the same weight, which is not confirmed by our findings, where great differences are observed in the OR of these two markers. In this context, a recent letter to the editor published by Bossuyt (42, 43) reported that the probability for RA using CCP2 ELISA (Euro-Diagnostica) increases from 3.4 to 73.6 (low versus 3× ULN value), which is significantly higher than reflected in the classification criteria (2 versus 3 points). This is somewhat contradictory to our findings where we saw an increase from 16.3 to 45.6 in the CCP2 OR at the cutoff and at the 3× ULN, respectively, and with regards to the CCP3 CIA where the OR increases from 107.5 to 192.5, which corresponds well to the weighting of the classification criteria. By contrast, the CCP3 ELISA did not show a significant increase. Future refinements of the RA classification criteria may attribute a higher relative weight to a high-positive ACPA compared with a low-positive ACPA. Future studies are needed to evaluate whether cutoff points for ACPA assays (especially for the 3× ULN) are aligned between different manufacturers.

Currently, the combination of ACPA with RF by turbidimetry which detects all RF isotypes is the most commonly used approach (44–46). In our study, the combination of CCP3 with RF IgM, on both platforms, showed a very high OR, higher than the OR with the individual markers at the cutoffs and equivalent to the CCP3 CIA OR at the 3× ULN. The addition of RF IgA to the CCP3 and RF IgM ELISA combination resulted in a higher OR. By contrast, the addition of RF IgG to this model did not seem to be valuable from a diagnostic perspective. Interestingly, the combination of both ACPA tests with different antigens (CCP2 and CCP3), did not give a better discrimination between RA and controls when compared with the models based on CCP3 only. However, a higher specificity was observed with the CCP2 and CCP3 ELISA combination compared with the individual CCP assays. These results are in agreement with what was recently published by Vos et al. (17), where the positivity for both CCP2 and CCP3 resulted in the most specific identification of the RA patients. Although the samples were tested for RF IgM in that study, this marker was not analyzed in combination with CCP. In addition, it has been previously reported that ACPA can be found in PsA patients with a prevalence between 5 and 13% (47–49) and that they are linked to erosive disease (49, 50). In our cohort, ACPA were detected in 13.1% of PsA patients with anti-CCP2, however, only in 1.3 and 1.7% of patients with the CCP3 IgG ELISA and CIA, respectively. This would suggest a higher specificity of the CCP3 assay compared with CCP2 in this group of patients. No data were available about erosive disease in PsA patients.

Besides the OR, it is also important to assess how many patients can be captured using a single test or a combination of tests. By combining test results, often the specificity and consequently the OR increase substantially. However, this usually in turn reduces the number of patients that can be correctly classified. Although the added value of the CCP3 and RF IgM combination versus the CCP3 individual assays is limited, a higher number of patients could be correctly classified with the combinatory model compared with the individual CCP3 assays, with fewer patients found within the areas on uncertainty (interval II) (Figures 2B,D). These data suggest a potential diagnostic utility of a combinatory model approach using combinations of biomarkers, especially RF IgM and anti-CCP3.

Even though ACPA have significantly contributed to improve the diagnosis of RA, there is an unquestionable need for novel biomarkers to enhance the early diagnosis of RA, especially in patients currently classified as seronegative, as well as, to define the different RA subclasses and to stratify patients according to different disease phenotypes (29). Once more diagnostically relevant biomarkers have been established, modern multianalyte techniques for the simultaneous detection of a wide spectrum of markers, may provide additional benefits in diagnosis, classification of RA subtypes, stratification of patients to start early treatment, and potentially lead to prevention. The inclusion of novel RA-associated markers in multiplex assays and prediction models may facilitate the use of profiling in diagnostic routine laboratories.

One limitation of this study was that ACPA and RF are used in the diagnosis of RA in clinical practice. This could have influenced the observed association between these antibodies and the predictive value of RA. This is an inherent problem of any study that investigates the performance of RF and ACPA in the diagnosis of RA. Furthermore, the findings of these studies would have to be validated in independent cohorts that include additional disease controls.

In the context of precision medicine, the combination of biomarkers represents a very promising tool to improve the diagnosis of RA patients (17) and to predict the possible association with disease development (51, 52) and therapeutic response (53).

## Conclusion

Anti-citrullinated peptide/protein antibodies and RF CIA showed good clinical performance in this large cohort. RF IgA performance is very platform dependent, with the CIA demonstrating superior performance compared with the ELISA. Overall, the performance of CCP3 was superior to CCP2, when analyzed individually as well as in combination with RF IgM. CCP2 and CCP3 complement each other and have a better predictive value than the individual assays but did not show a better discrimination between RA and controls. The combination of several of these biomarkers, in particular CCP3 IgG and RF IgM, seems to be useful for the clinical diagnosis of RA patients and to help correctly classify a higher number of patients.

## Author Contributions

LM-P performed the statistical analyses and drafted the manuscript. MN designed the study, helped with the statistical analyses, and drafted the manuscript. LM-P and MN contributed equally to this paper. CL and PR-L helped design the study. CL collected and managed the samples. PR-L performed the CCP2 testing. CB designed the study. LC carried out the CCP3 and RF testing and compiled the results. CG designed the study. MM designed the study and reviewed the statistical analyses. All authors read, reviewed, and approved the final manuscript.

## Conflict of Interest Statement

LM-P, CB, and MM are employed at Inova Diagnostics, Inc. LC was previously employed at Inova Diagnostics, Inc. MN, CL, PL-R, and CG have no conflict of interest to disclose.

## Abbreviations

RA, rheumatoid arthritis; AS, ankylosing spondylitis; axSpA, axial spondyloarthritis; ASAS, Assessment of Spondyloarthritis International Society; PsA, psoriatic arthritis; CASPAR, classification criteria for psoriatic arthritis; ACPA, anti-citrullinated peptide/protein antibodies; CCP, cyclic citrullinated peptide; RF, rheumatoid factor; ELISA, enzyme-linked immunosorbent assay; CIA, chemiluminescent immunoassay; QF, QUANTA Flash^®^; QL, QUANTA Lite^®^; AUC, area under the curve; ROC, receiver-operating characteristic; FDR, false discovery rate; ACR, American College of Rheumatology; EULAR, European League Against Rheumatism; CU, chemiluminescent units; RLU, relative light units; ULN, upper limit of normal; OR, odds ratio; RUO, research use only.
