# Supplementary figures and images for: Comparison of Serological Biomarkers in Rheumatoid Arthritis and Their Combination to Improve Diagnostic Performance
Source: Front Immunol. 2018 Jun 6;9:1113. doi: 10.3389/fimmu.2018.01113 (PMC5997814; doi:10.3389/fimmu.2018.01113)

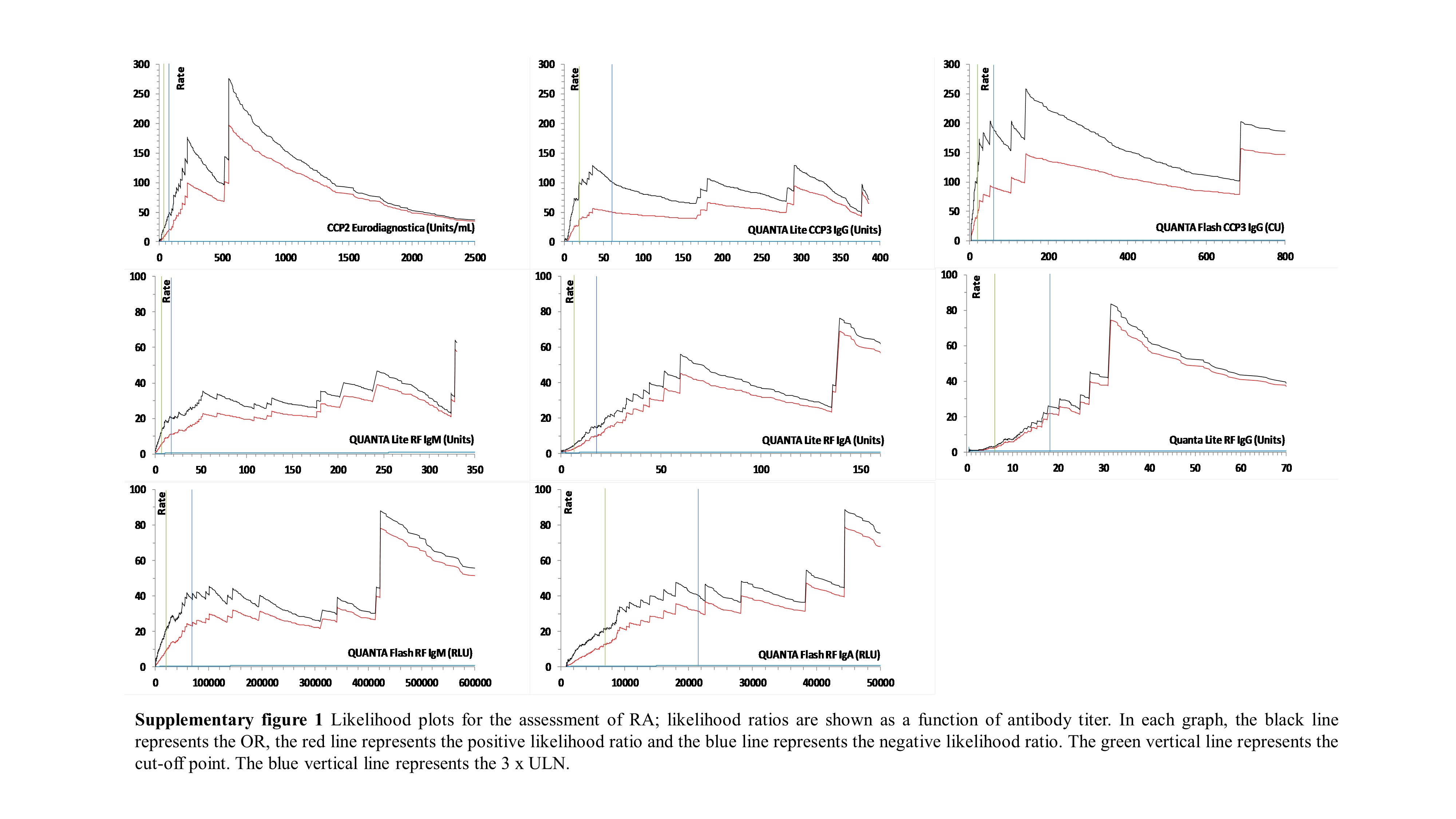

Supplement: Supplementary file 1 [file image_1.jpeg]
