# Supplementary material for: Comparison of Serological Biomarkers in Rheumatoid Arthritis and Their Combination to Improve Diagnostic Performance
Source: Front Immunol. 2018 Jun 6;9:1113. doi: 10.3389/fimmu.2018.01113 (PMC5997814; doi:10.3389/fimmu.2018.01113)
Supplement: Supplementary file 2 [file Table_1.docx]

Supplementary Material

Comparison of serological biomarkers in rheumatoid arthritis and their combination to improve diagnostic performance

**Laura Martinez-Prat^+^, Michael J. Nissen^+^, Céline Lamacchia, Chelsea Bentow, Laura Cesana, Pascale Roux-Lombard, Cem Gabay, Michael Mahler*.**

^+^Note: Laura Martinez-Prat and Michael J. Nissen contributed equally.

*** Correspondence:** Dr. Michael Mahler: mmahler@inovadx.com or [m.mahler.job@web.de](mailto:m.mahler.job@web.de)

## Supplementary table

**Supplementary table 1** Summary of the characteristics of the assays.

|  | **CCP2** | **CCP3** | **RF** | | | | | |
| --- | --- | --- | --- | --- | --- | --- | --- | --- |
| **Assay** | **CCP2 IgG ELISA** | **CCP3 IgG CIA** | **CCP3 IgG ELISA** | **RF IgM CIA** | **RF IgM ELISA** | **RF IgA CIA** | **RF IgA ELISA** | **RF IgG ELISA** |
| **Manufacturer** | Euro Diagnostica | Inova Diagnostics | Inova Diagnostics | Inova Diagnostics | Inova Diagnostics | Inova Diagnostics | Inova Diagnostics | Inova Diagnostics |
| **Technology** | ELISA | CIA | ELISA | CIA | ELISA | CIA | ELISA | ELISA |
| **Units** | U/mL | CU | Unit | RLU | Unit | RLU | Unit | Unit |
| **RUO** | NO | NO | NO | Yes | No | Yes | No | No |
| **Determinations per kit** | 96 | 100 | 96 | 100 | 96 | 100 | 96 | 96 |
| **Assay time** | 90 min | 30 min | 90 min | 30 min | 90 min | 30 min | 90 min | 90 min |
| **Solid phase** | ELISA well | Paramagnetic microparticles | ELISA well | Paramagnetic microparticles | ELISA well | Paramagnetic microparticles | ELISA well | ELISA well |
| **Conjugate** | HRP conjugated goat anti-human IgG | Isoluminol conjugated anti-human IgG | HRP conjugated goat anti-human IgG | Isoluminol conjugated anti-human IgM | HRP conjugated goat anti-human IgM | Isoluminol conjugated anti-human IgA | HRP conjugated goat anti-human IgA | HRP conjugated rabbit anti-human IgG |
| **Calibration frequency** | Every time the assay is run | Once per reagent lot | Every time the assay is run | Once per reagent lot | Every time the assay is run | Once per reagent lot | Every time the assay is run | Every time the assay is run |
| **Results interpretation (cut-off)** | 25 U/mL | 20 CU | 20 Units | 10,344 RLU (preliminary) | 6 Units | 7425 RLU (preliminary) | 6 Units | 6 Units |
| **Matrices** | Serum, citrated or EDTA Plasma | Serum | Serum, citrated or EDTA Plasma | Serum | Serum, citrated or EDTA Plasma | Serum | Serum, citrated or EDTA Plasma | Serum, citrated or EDTA Plasma |
| **Sera volume required** | 5 μl | 10 μl | 5 μl | 10 μl | 5 μl | 10 μl | 5 μl | 5 μl |
| **Isotype** | IgG | IgG | IgG | IgM | IgM | IgA | IgA | IgG |

CCP= Cyclic Citrullinated Peptide; RF= Rheumatoid Factor; CIA= Chemiluminescent Immunoassay; RUO= Research Use Only, CU= Chemiluminescent Units, RLU= Relative Light Units.
